# Friendship: a phenomenological exploration of the lived experience of youth and of peer support workers in Youth FACT (flexible assertive community treatment) services

**DOI:** 10.1080/17482631.2026.2660040

**Published:** 2026-04-16

**Authors:** Hege Arvesen, Nina Helen Mjøsund, Vibeke Krane, Mona Sommer

**Affiliations:** a University of Southeastern Norway, Faculty of Health and Social Sciences, Department of Health, Social and Welfare Studies, Drammen, Norway; b Head of the Department for Research and Development, Manager of Research in the Division for Mental Health and Addiction, Vestre Viken Hospital Trust, Drammen, Norway

**Keywords:** Friendship, mental health, mental health care, peer support workers, young people, hermeneutic phenomenology, relational approach, Youth FACT

## Abstract

**Background:**

Friendship is recognized as vital for mental health and quality of life. It encompasses both protective and challenging aspects, constituting a relational and existential dimension in human life. Despite extensive research on the significance of friendship, less is known about how friendship is experienced in everyday life among individuals facing mental health challenges.

**Aim:**

To explore the lived experiences of friendship as described by young people receiving follow-up from Youth FACT and peer support workers employed in these services.

**Method:**

A hermeneutic phenomenological approach inspired by van Manen was employed. Semi-structured in-depth interviews with 7 young people (aged 15−18) and 7 peer support workers (aged 28−45) were analyzed. Analysis involved the selection of rich, experience-near descriptions, their transformation into experiential anecdotes, and reflective, interpretive exploration.

**Findings and concluding remarks:**

Friendship reveals as a complex, living, existential phenomenon, integral, continuously in motion as dynamic processes beyond our control. Friendship offers possibilities without guarantees, unfolding through presence, vulnerability, movement, and freedom. Among young people and peer support workers in Youth FACT, our exploration show how layered meanings of friendship uncover uniquely in each encounter, protective for some, burdensome for others, and at times both within the same relationship and experience.

## Introduction

Friendship represents a universal human phenomenon that is part of our lifeworld, manifest in close lasting friendships, fleeting friendships, dreams of having friends, or the experience of solitude. When complex mental health challenges disrupt school, work, family, and leisure, relational difficulties in everyday social life become particularly evident (Bjornestad et al., 2017; Boydell et al., 2002; Cleary et al., 2018). In this article, to illuminate these different aspects of friendship, we explore the lived experiences of youth and peer support workers.

From kindergarten through school, children and young people are expected to form friendships, belong, and function socially. Friendship supports not only learning and coping but also mental health and quality of life (FHI, 2025; Gotting et al., 2025; Norwegian Directorate of Health, 2024; Parker & Asher, 1993; Salvas et al., 2025). Friendship is recognised as a key psychological and social resource in the lives of children and young people. Accordingly, schools and kindergartens in Norway implement systematic measures intended to promote a sense of belonging, interaction, and social skills (FHI, 2025; Norwegian Directorate of Health, 2024).

During adolescence, when identity, belonging, and self-understanding are being developed, friendship becomes especially vital (Bukowski et al., 2020; Manchanda et al., 2023; Shah et al., 2024). For many young people, adolescence is a stage where the foundation for mental health, relationships, and future quality of life is established (FHI, 2025; Norwegian Directorate of Health, 2024). Population studies link close and stable friendships to lower mental health risks, greater social resilience, and better developmental outcomes (Alsarrani et al., 2022; Gariépy et al., 2016; Spithoven et al., 2017). Qualitative and quantitative research involving young people with mental health challenges shows that friendship can offer hope, motivation, and escape from isolation during difficult life phases, such as psychosis episodes (Bjornestad et al., 2017; Skou Storm et al., 2025). King et al. (2016) describe close friendships as a “psychological vaccine,” based on quantitative longitudinal data showing that the protective effect is connected to emotional support and social integration. Shah et al. (2024) conducted a quantitative study confirming that friendship serves as a buffer against stress and loneliness, especially during transitional periods. For some young people, social withdrawal may feel like necessary protection (Boydell et al., 2002).

However, as with all forms of human relationships, friendships also entail relational challenges. For youth facing mental health challenges, relational difficulties, conflicts, rejection, and social withdrawal can turn friendship into a source of added stress, shame, isolation, and loneliness, as shown in both qualitative and quantitative mental health studies (Boydell et al., 2002; Cleary et al., 2018; Spithoven et al., 2017). Friendship can also involve conflicts, imbalance, and vulnerability (Alsarrani et al., 2022; Lahad & van Hooff, 2023; Spithoven et al., 2017). Young people with mental health issues may experience relational struggles related to friendship, which can be a risk factor for mental health (Alsarrani et al., 2022; Cleary et al., 2018; Dunbar, 2025; Gariépy et al., 2016; Manchanda et al., 2023; Spithoven et al., 2017). Additionally, qualitative studies of lived experience show that young people facing mental health challenges may withdraw socially as a protective measure, which can worsen feelings of loneliness and mental discomfort (Boydell et al., 2002). Thus, friendship embodies both protective and challenging dimensions, as a relational and existential phenomenon. Despite extensive research on the importance of friendship, less is known about how it is experienced and lived in daily life amid complex mental health challenges, especially when young people face impairments in multiple areas of functioning.

### Purpose

The purpose of this study is to explore how friendship is experienced and acquires meaning in the everyday lives of young people, receiving services from Youth FACT and peer support workers employed there. Inspired by a hermeneutic-phenomenological approach, we aim to illuminate the complexity of friendship as a lived phenomenon grounded in subjective experiences, particularly within contexts of mental health struggles. The research question is: “*How is friendship experienced within the lifeworld of young people and peer support workers in Youth FACT?”*

## Methods

The study is anchored in a hermeneutic phenomenological approach, inspired by van Manen (van Manen, 2017, van Manen, 2023). This approach, called “phenomenology of practice,” seeks to uncover the essential meanings of lived experiences as pre-reflective, bodily, and relational moments that constitute people's lifeworld. The approach employs reflective writing that stays close to how phenomena present themselves in everyday life (van Manen, 2023). In practice, this occurs as the researcher moves between openness and wonder toward the phenomenon under investigation and active interpretation of meanings that emerge in people's lifeworld (van Manen, 2023). In this way, research becomes a dialogical process where the researcher's pre-understanding plays a role in meaning-making, as all understanding occurs in interplay between the researcher's perspectives and the phenomenon (van Manen, 2023, van Manen, 2016).

The concept of lifeworld (Lebenswelt) refers to the everyday world we live in before it is theorised or objectified. In van Manen, (2016, 2023), the lifeworld serves as a central phenomenological starting point for understanding human experience as it presents itself in concrete situations. It is described through four existentials that always characterise our being-in-the-world: temporality (how we experience the rhythm of life), spatiality (the significance of place and our situatedness), corporality (the bodily and sensory way we are present), and relationality (our being-with-others). These dimensions provide a framework for exploring how phenomena—such as friendship—are experienced and gain meaning in people's everyday lives. Van Manen emphasises that phenomena do not have one objective truth but can be understood through multiple layers of meaning (van Manen, 2016). Our phenomenological approach, inspired by van Manen (2023), seeks to attend to the lived experience as it was immediately experienced in the moment of its occurrence. We recognise, however, that such an effort is ultimately unattainable, as any attempt to describe lived experience is always made in retrospect, shaped by recollection, reflection, and interpretation. Nevertheless, it is precisely this methodological aspiration that animates phenomenological enquiry: to come as close as possible to the immediacy of lived meaning—to return to the experience itself, before it is conceptualised or theorised.

Hermeneutic phenomenology as a method allows us to explore the complexity of lived experience, in which experiences are nuanced, multifaceted, and relational. The method establishes an ethical and philosophical framework that enables a research practice characterised by hermeneutic sensitivity and respect for the lived experience (van Manen, 2016, 2017, 2023). In this study on friendship, this means approaching the phenomenon as an existential dimension in human life.

## Collaborative approach

The study builds on a scientific approach that gives experienced-based knowledge a central place in the research process (Beresford, 2019; Borg et al., 2012). Collaborative research ensures that diverse perspectives and lived experiences are integrated and valued in knowledge development. To realise this, a competence group consisting of five individuals with experiences of mental health and/or substance use challenges and follow-up from health services has been established. The members of the competence group are not part of the study's sample.

The competence group served an important advisory and consultative role in shaping the study (Krane et al., 2025). In this capacity, they contributed to central parts of the research process, including recruitment, development of the interview guide, and sharing their perspectives on preliminary findings (analysis) and discussion relevance. Specifically, they provided input on the interview guide by assessing whether questions were understandable, contributed to the recruitment process by giving feedback on the language and comprehensibility of consent forms, shared their understandings of preliminary friendship findings to assess personal resonance and gave feedback on the discussion section to ensure relevance for recovery-oriented practice.

This dialogical approach makes knowledge development a process where researchers and individuals with experienced-based knowledge come together (Krane et al., 2025). Members of the competence group contribute insights based on whether it resonates with their own experiences. This type of collaboration can help strengthen the study's relevance, as individuals with lived experience possess a unique competence that adds valuable perspectives to the research, opens up new insights, mutual learning, and polyphony in the interpretation of the material, thereby enhancing the study's epistemological breadth (Krane et al., 2025). The purpose of a collaborative approach, in this particular study, is to expand the understanding of the phenomenon of friendship, while reflecting on which voices and experiences are granted space, validity, and value in knowledge development (Sommer, 2019).

## Study context: Youth FACT

Youth FACT is a multidisciplinary and outreach health service targeted at young people aged 12 to 18 years (up to 25 years if needed) with complex mental health and/or substance use challenges (Ministry of Health & Care Services, 2023; Nord-Baade, 2022). The service integrates expertise from specialist mental healthcare, municipal health services, and peer workers to ensure holistic and integrated support for the target group (Landheim & Odden, 2020; Nord-Baade, 2022). The target group includes young people who have not benefited sufficiently from ordinary support services and who, for various reasons, isolate themselves, are reluctant to attend school, abuse substances, are involved in criminal activity, have social difficulties, or struggle with complex mental health challenges (Nord-Baade, 2022). In this study, the term *mental health challenges* is used in accordance with recovery-oriented language practices. Within Youth FACT, the term promotes a person-centred understanding that highlights individuals’ holistic experiences of what they are struggling with, without necessarily framing these experiences as illness or diagnosis (Nord-Baade, 2022). This does not exclude the possibility that *mental health challenges* may also include psychiatric diagnoses where relevant.

Youth FACT teams include professionals such as psychiatrists, psychologists, social workers, social educators, nurses, vocational specialists, and peer support workers. The teams work holistically and flexibly to tailor support to each young person's needs, resources, and wishes (Nord-Baade, 2022). The teams operate according to recovery-oriented principles with particular emphasis on the importance of social networks and social arenas. Working in a recovery-oriented manner means understanding recovery as a personal, social, and relational life process, where belonging, identity, hope, and participation are central dimensions (Anthony, 1993). Relationships with family, friends, and networks are highlighted as equally important as formal treatment measures for recovery processes (Tew et al., 2012; Tew, 2004; Topor et al., 2011).

The peer support worker in the team is employed based on their own experiences with mental health and/or substance use challenges and is a fixed part of the interdisciplinary work. All Youth FACT teams must have at least one peer support worker (Nord-Baade, 2022; Norwegian Directorate of Health, 2022). The role involves contributing experienced-based knowledge on equal footing with professional expertise and functioning as a bridge-builder, supporter, and resource person for youth, families, and other agencies. Peer support workers actively participate in the team's daily practice and engage in outreach work in young people's arenas, such as schools, leisure environments, and local communities (Åkerblom & Mohn-Haugen, 2023; Nord-Baade, 2022). Their role can provide insight, hope, and security for young people and can offer practical advice based on personal knowledge of recovery processes (Gillard et al., 2024; Gillard et al., 2022; Høiseth et al., 2024; Viking et al., 2022).

## Participants and recruitment

Information about the project was conveyed through Youth FACT team leaders across Norway, who actively supported recruitment from established teams. Recruitment occurred in two stages. First, team leaders facilitated contact with peer support workers, with whom the first author made direct arrangements. Subsequently, both team leaders and peer support workers, who knew the young people best, identified suitable young participants and facilitated contact with the first author. Newly established teams were excluded to ensure participants had sufficient experience with the service. A total of 14 people participated, divided into two groups.

The first group consisted of seven young people aged 15 to 18 (4 boys, 1 girl, 2 nonbinary) receiving Youth FACT follow-up. Their challenges varied, but all qualified for Youth FACT services due to significant functional impairment, including school refusal, criminality, substance use, anxiety, isolation, social exclusion, and/or loneliness.

The second group consisted of seven peer support workers aged 28 to 45 (5 men, 2 women) working in Youth FACT services. They were employed based on experienced-based knowledge gained from their own lived mental health challenges, which varied and included eating disorders, anxiety, substance use, criminality, trauma, or experiences as next of kin facing such challenges.

## Data collection method

The data material consists of fourteen semi-structured individual interviews. While semi-structured interviews are not inherently aligned with hermeneutic-phenomenological research, we followed a phenomenological orientation by directing follow-up questions toward participants' lived experiences of friendship in everyday life (e.g., “Can you describe an experience?” rather than “Can you tell me about an experience?”). This flexibility allowed us to pursue unexpected and important aspects in the participants' descriptions of the phenomenon. This methodological approach aligns with hermeneutic-phenomenological methodology in which the dialogue between researcher and participant is a fundamental part of uncovering meaning in lived experiences (van Manen, 2023). Prior to the interviews, we developed tailored interview guides with questions that gave participants room to recount their experiences.

## Analysis process

As the first step in the analysis process, we began by reading the data material in its entirety, with an open attitude to form an overall impression consistent with van Manen's descriptions of the analysis work (van Manen, 2023). We asked ourselves: “What are the participants telling us about the phenomenon of friendship?” and marked this in the data material.

We then went back to the individual interviews to explore the lived meaning in participants' experiences and to get as close as possible to the experience of friendship (van Manen, 2023). Here, we asked ourselves questions such as: *“How do participants describe their experiences of friendship in everyday life?” “What words do participants use to describe friendship?” “Which descriptions show us experiences of friendship?”* This focus on lived experiences served as a guiding orientation throughout the analytical process.

Next, we collected quotes from the interviews we considered potentially suitable for phenomenological exploration. This yielded rich material that we further processed by compressing quotes into concise, readable excerpts. These excerpts were shaped into experiential anecdotes. In van Manen's method, anecdotes are concrete descriptions of lived experience, such as particular situations, relational encounters, or meaningful moments, that serve as a methodological tool for phenomenological reflection. Through their vivid, concrete details, anecdotes create an evocative contact with the phenomenon. Evocative here refers to language that poetically calls forth and intensifies the reader's pre-reflective understanding of the lived meaning. This aims to open up new insights and provide a basis for exploring friendship's essential structures in hermeneutic phenomenological research (van Manen, 2023).

Repeatedly working with and through the compressed anecdotes allowed essential aspects of friendship, as described by the participants, to emerge, providing an analytical foundation for even deeper exploration of the phenomenon. The purpose here was to *show*, through interpretation of the anecdotes, how friendship appears and presents itself from the participants' perspective. This process also involved a continuous movement between data material and text processing, creating a dynamic, living analysis process characterised by reflection and new insights. van Manen (2023) emphasises writing and rewriting as central tools in phenomenological analysis and reflection. Writing is not merely a means of conveying text but a meaningful part of the research process where understanding and meaning gradually unfold (van Manen, 2023). We wrote, rewrote, and exchanged meanings among the authors, received feedback from the competence group, and kept circling back to the data over and over. This iterative reflective process led us to a deeper unfolding of meaning in the text. The anecdotes and their interpretations are presented as findings in this article.

## Ethical considerations

Prior to data collection, the project was reviewed and approved in accordance with current data protection guidelines by the Norwegian Agency for Shared Services in Education (case no.: 698643) and current ethical guidelines by the Regional Committees for Medical and Health Research Ethics (case no.: 934245). All participants volunteered and provided written informed consent prior to the interviews.

Data privacy was ensured by recording all interviews, transcribing them verbatim, anonymizing them, and storing them in a secure zone in accordance with data protection guidelines. All participants are anonymized by being assigned a code in the transcribed material known only to the first author. They have been given pseudonyms: Ada, Marie, Nana, and William, and gender is indicated when presented in this study.

Special considerations were taken in both the recruitment process and the interviews, as we can assume that the topic of friendship may evoke unforeseen emotions that some participants may find challenging. In addition, we considered the young participants vulnerable in research due to their struggles with complex mental health challenges for various reasons. Our assessments are based on the Declaration of Helsinki (2024) and the Health Research Act (2009), which emphasise the protection of vulnerable groups in qualitative research. We implemented various research measures to reduce the burden on participants: Leaders in Youth FACT teams assisted in assessing and inviting young participants and forwarding our invitation to participate to peer support workers with capacity. We arranged the interviews so that time, place, and manner aligned with participants' preferences. We also allowed young participants to bring a trusted person, and several did this. Furthermore, we planned a follow-up to ensure that young participants had contact persons familiar with the study in case the interview was experienced as burdensome. This information was clarified with the young participants before the interview concluded. We assessed the peer support workers as less vulnerable than the youth participants but, nevertheless, we encouraged them to contact their team leader if they needed to discuss any aspects of the interviews they conducted.

## Findings

Shared presence in the emergence of friendship Ada is a peer support worker, working in a Youth FACT team. When she was younger, she was often scared and alone. She mostly sat by herself in the schoolyard. She witnessed friends playing, smiling, and laughing without participating herself. She wanted a friend but did not know how to make one. This made Ada feel she had no value. When Ada met Thea, a change in her lifeworld happened, as she experienced a presence and community that she had previously thought unattainable.

Thea approaches me with determined steps. Our gazes find each other amid the crowd. We haven't planned it. It simply happens. She sits close beside me on the wall. It's recess. Conversation flows effortlessly between us. She is engaged, eager. I smile as I listen to her story. I respond, try to say something funny. Thea laughs, talking with me, filling me with a bubbling joy and hope. For the first time, I can trust that someone wants to be with me. It suddenly strikes me why people have friends. Here beside Thea on the wall, I realise I have gained a friend.

The experience of friendship is not merely a thought or feeling, but a bodily encounter of being close to another human being. In the nearness on the wall, something more than physical proximity arises, a quiet assurance that being together holds meaning beyond the shared moment. Thea sees Ada, affirms her, seeks her out, and offers a piece of herself to Ada. Small everyday signals that friendship exists and that Ada is not alone. Their conversation flows lightly between them, unfolding this presence as Ada dares to share in return, drawn closer as an equal in the space of friendship alongside Thea.

It is in this nearness, this attentive presence, this shimmer of being seen and affirmed, that they dwell together. The laughter ripples between them, light and unforced. No roles weigh down their being-together, no expectations, no duties, only the quiet rhythm of two who choose each other's company. Each glance, each shared breath on the wall, speaks this choice through the body's own language, and their friendship takes bodily form as a living assurance of being desired, of mattering, just as they are.

Thea sitting close beside her on the wall, presence surges through Ada's body like bubbling effervescence lifting her toward the world. Laughter flows effortlessly between them, Ada's smile meeting Ada's attention. In this presence, the choice to sit there, to listen, to laugh, breathes quiet strength into Ada, a readiness she carries forward from the wall.

The schoolyard echoes still linger. Ada's experiences of sitting alone, watching others play and laugh, and feeling herself fade into worthlessness. What once felt like existential unease, doubts of belonging, of value, becomes a shimmering hope, alive in her. Friendship dwells here, fragile and irreplaceable, taking bodily form in the space between them. In this shimmer of being chosen, Ada senses why people seek friends. Now Thea sits beside her, and that emptiness transforms.

### Shared vulnerability as lived friendship

Marie is a peer support worker in a Youth FACT team. As a young person, she struggled with mental health challenges. Despite these struggles and her limited capacity to maintain contact with friends during this period, she experienced that her friends stayed with her and continued to reach out even when she could not.

Every afternoon, I hear Linda's tentative knock on the door. A small signal through the silence. I am lying under the duvet, with only the light seeping in through the gap in the curtains. Linda tiptoes in on sock feet and sits down on the chair by the window. She asks how I am. I mumble a faint “fine,” though I can barely manage. In the dim light, she finds the book and begins to read. She reads with intention, immersed in the book. Her voice clear and steady. Gratitude builds within me for this time with Linda. A quiet admiration that she keeps coming. I can't really follow along. I keep thinking what poor company I must be, lying here in bed. Yet she reads and has done so for many days in a row. She reads for us both. Marie is alone in her room, under the duvet, with only herself for company. Vulnerability thickens the air, present yet unseen and unnoticed. When life rolls in heavy waves, the lifeworld contracts inward.

Linda's tentative knock breaks the silence like a small signal through the heavy stillness. The duvet clings like fragile protection; body heavy, words trapped. In the dim light seeping through the curtain gap, Linda finds the books. Linda's voice cuts through the room, clear and steady, reaching Marie's vulnerability with presence. In this moment, vulnerability appeared as shared. Linda enters the contracted space, bearing the moment’s relational weight through her steady voice. Marie rests within it. Their friendship endures through this gentle imbalance, patiently alive.

Shared vulnerability manifests not in words or equal exchange, but in actions that hold space amid unevenness. Even when Marie knows she offers poor company, unable to follow or engage, Linda`s knock returns day after day, her voice filling the room. Both choose persistence despite the imbalance. Marie withdraws and is carried; Linda offers presence without demanding reciprocity. Vulnerability seems to shimmer here as mutual acceptance, without demands for perfection, with willingness to bear uneven loads together.

The unpredictable rhythm of friendship Nana receives follow-up from the Youth FACT team. Her upbringing was marked by turbulence, unrest, and insecurity. At 14, she moved to an institution but now lives independently. Angela is her best friend. Nana experiences this friendship as deeply valuable, but conflicts sometimes arise that linger. During these periods, thoughts emerge that she is unworthy of friendship. One such conflict unfolds on the way to the leisure club:

Angela and I head to the leisure club together, bickering the whole way. A gnawing restlessness scrapes under the skin. The air between us hangs heavy. Words sharp. At the club, Angela glides effortlessly among the other friends. She laughs, smiles lightly at them. I'm there, but the unrest in my body roars like a deafening noise. I keep my distance from the others. Exhausted. Most of all, I want to be alone, but I have nowhere to go. Angela says something to me. I snap back, irritated. Her gaze locks on mine: “What is wrong with you, Nana? Do you have to be so grumpy?” Her words tip me over the edge. We argue loudly, out of control. I turn and walk away. Into the darkness. Away from everything and everyone. I can't take it. There's nowhere to rest.

Dwelling into Nana’s subjective experiences, it seems like lived experiences of friendship are not a state, but a movement. A lived relation unfolding in time and space, in body and mind, in words and silence. It is a way of being together that cannot be planned, controlled, or guaranteed. Nana's experience shows us that friendship is a living process, not necessarily a safe structure. It is shaped in what happens and in what does not happen; in gazes that meet and gazes that avoid, in words spoken and words left unsaid. It is in this unpredictable interplay that friendship takes form and loses it again. The friendship between Nana and Angela is not static. It lives, moves, and changes. Nana carries a past marked by insecurity and rupture. A past that lives on in her body, her tightening gaze, her sharp responses. When unrest rises in her, it is not merely a feeling. It takes bodily form in her way of being toward Angela. Friendship does not always tolerate the unrest, nor always bear the weight of the unspoken. Angela's question: “Do you have to be so grumpy?” arrives not merely as words, but as a lived touchpoint in their friendship. It marks where one friend's unrest meets the other's limit. Friendship here reveals its texture: not an endless tolerance, but a relation that frays at the edges of what each can bear. Nana's withdrawal into darkness becomes the space where this fraying shows itself most clearly. Not just physical distance, but a contraction of the shared lifeworld, where being-with strains against being-alone.

### The challenge of freedom in friendship

William is a youth receiving follow-up from the Youth FACT team. He has experienced a turbulent upbringing and lives in an institution. He tries to keep a distance from Nick, his best friend, and the environment he was previously part of. This is an active choice on his part, a kind of life path decision for the future.

‘I can't have contact with you right now.’ That was the last message I sent Nick. I need to step back. We've gotten ourselves into so many difficult situations together. There's a reason I'm where I am now. I sent that message to Nick last week. It's best this way, that we don't talk. Something deep inside me stirs. Sadness bubbles to the surface. The solidarity we shared, the hours spent in the room, the laughter, the humour. I look down at my phone. I resist the urge to send him a message to hear what he's up to. I miss the feeling of us against the world. But now I want to be part of the world.

Freedom in friendship unfolds in the fragile space between closeness and distance. For William, freedom appears in his inner world as a choice to engage or withdraw, to connect or stay away from Nick. Freedom in friendship lives as unreflected nearness until demand reflection. When William sends Nick the message, “I can't have contact with you right now,“ the decision both closes and opens doors.

Thus, freedom is not merely freedom from something, but to a greater extent freedom *to* something; to shape life and find paths leading us forward. It may seem like a risk with no promises, a heavy lift, woven with uncertainty at every step. William's experience reveals freedom's duality; that freedom is always accompanied by vulnerability, reminding us that the lived space of friendship is both open and fragile.

Freedom weighs on William as heavily as it lifts him, felt in his body and filling him with emotion. There is a deep stirring inside that pulls him to reflect on his choice. Doubt is creeping in, and the feeling that once had meaning is fading away. Sadness wells up inside him. Memories of shared laughter and solidarity surface, sharp against the difficult situation that led him here. Staying connected with Nick won't push him forward. Yet, the bonds pull in the opposite direction.

Caught in this swing, William senses freedom’s fragility presses close. The decision felt right then and still does. Still, even now, he balances between clinging to the past and stepping into the unknown ahead. Freedom swings like a pendulum inside him, shifting between what he knows and what he feels. Choosing distance means opening oneself to longing, to doubt, to the quiet pain of letting go of what has been meaningful. Freedom in friendship is not only a privilege, but also a risk. William has no guarantees for how his choice will shape his life. It leaves him vulnerable.

### Discussion friendship's existential dimensions - phenomenological insights

The purpose of this study was to explore the phenomenon of friendship as it manifests in the participants’ lifeworld. Inspired by hermeneutic phenomenology, we remain dedicated to capturing participants' pre-reflective experiences of friendship as they appear in the lifeworld. This methodological stance enabled us to stay close to participants' pre-reflective, lived experiences of friendship. In this discussion, we build on the findings to illuminate the complexity of friendship as a subjective, embodied experience, and relate this to mental health work and recovery-oriented practices.

In our study, friendship appears as an intertwining of internal existential processes and external social encounters, woven together in lived experiences, constantly in motion, as a dynamic process beyond our control. Friendship comes without guarantees but with possibilities, and its complexity shows itself through aspects such as presence, vulnerability, movement, tension and freedom. This resonates with van Manen (2023) notion of lived relationality as inherently open, situated, and unfinished. Merleau-Ponty (2013) phenomenology further underscores the body's role in experience: we do not stand outside friendship as observers but live it bodily and situated in time and space, directed towards others whom we can never fully grasp. Relationships are therefore always, to some extent, open and unpredictable (Merleau-Ponty, 1945).

Seen in this light, friendship exemplifies how lived experience integrates the existential dimensions of being-in-the-world, where identity, belonging, and meaning emerge through our embodied relatedness to others (van Manen, 2023). Our findings indicate that friendship constitutes a way of being in the world that touches how we understand ourselves to be, how we feel we belong, and what makes life meaningful. At the same time, the unpredictability that characterises friendships is not only a source of strain but also of possibilities. Considering through van Manen’s lifeworld existentials, what we carry with us of vulnerability and strength shapes the relational space (van Manen, 2016, 2023), where mental struggles can appear as a disturbance, yet also open up room for meaning-making and growth.

### Friendship's contextual meanings in mental health challenges

There is a broad understanding that friendship holds significance for mental health and well-being (Alsarrani et al., 2022; Boydell et al., 2002; Cleary et al., 2018; Dunbar, 2025; Gotting et al., 2025; King et al., 2016; Skou Storm et al., 2025; Spithoven et al., 2017) In mental health work, friendship is often highlighted as a relational and social resource that can protect against depressive symptoms and isolation, and at the same time promote health (Manchanda et al., 2023). Although friendship is presented as a central value in models, reports, and research (Alsarrani et al., 2022; Dunbar, 2025; FHI, 2025; Manchanda et al., 2023), its lived and experiential aspects of friendship often remain hidden in our unreflective, taken-for-granted ways of being in friendship. Surveys and manuals may record numbers of contacts or social activities, yet they reveal little about how friendship is *experienced* as part of the human lifeworld. While prior studies acknowledge that friendship's protective value varies between individuals, our insights reveal how this value emerges contextually in every-day-life among young people living with mental health challenges. For instance, Marie withdrew under her duvet when vulnerability thickened the air, while others found hope in shared presence during turbulent times. For some, friendship offers protection; for others, it brings strain. Often, these protective and challenging qualities co-exist in a single relationship, as seen in the experiences of Nana or William. Such experiences continually shape meaning-making in each singular moment. Our findings extend this understanding by showing how friendship's unpredictability, it’s movements of presence, vulnerability, and freedom, coexists with tensions of rejection and withdrawal. Participants described moments where daring to share oneself opened possibilities, yet the freedom to withdraw also preserved fragile relational space. This dynamic reflects what Boydell et al. (2002) identify as a central relational tension for people with mental health challenges as an ongoing struggle between the desire for connection and the simultaneous need to withdraw to protect one’s own vulnerability. This movement between approach and retreat resonates with van Manen (2023) lifeworld existentials, particularly lived relations and lived temporality. Here, mental health struggles simultaneously disrupt and enable growth in relational spaces.

Spithoven et al. (2017) observe the non-linearity of friendship's protective effects across youth, while Skou Storm et al. (2025) how friendships carry layered meanings in young adults' recovery-processes, serving as both resources and challenges, depending on lived context. Our experience-near insights from this study from Youth FACT settings reveal friendship as woven into subjective mental health processes, unpredictable yet rich in possibilities. These insights call for relational attention that moves beyond fixed structures and predefined models of care, toward a lived context-sensitive understanding of friendship.

### Implications for recovery-oriented practices

Based on our findings, subjective understanding and mental health are woven into the experience of friendship and become part of what continuously shapes the lifeworld. To be recognised and supported, this process requires experience-near and relational attention within recovery-oriented services. In the study context, recovery is understood as a holistic process where belonging, hope, and identity are central (Anthony, 1993; Leamy et al., 2011). Within this approach, social inclusion and network building remains areas in need of strengthening (Tew et al., 2012; Topor et al., 2011). A phenomenological understanding of friendship can contribute to this effort by illuminating how friendship experiences unfold in the everyday lives of young people struggling with mental health challenges.

In Youth FACT, recovery work becomes tangible through outreach on young people’s own terms, supporting participation in meaningful social arenas such as school, work, leisure, and local community-life. Here, different professionals collaborate with family, friends, and services around the young person (Broersen et al., 2024; Nord-Baade, 2022). Such recovery-oriented practices facilitate access to social arenas and create openings for friendship to emerge.

Our study examined experiences of friendship among both young people and peer support workers. Peer support workers contribute experienced-based knowledge through their close presence with youth in everyday follow-up (Nord-Baade, 2022). Their role opens space for navigating social landscapes together through an experience-near and relational manner. These encounters show how friendship can unfold through moments of shared space, relational curiosity, and time spent together, revealing both its enriching and challenging dimensions.

Yet, recovery-oriented practices also calls for attention to the existential dimensions of friendship: what it feels like to be in a relation when mental struggles are part of everyday life, including how these struggles shape experiences of friendship. Johansen et al. (2024) argue for a stronger focus on relational approaches to meet young people’s needs in Youth FACT services. Our findings echo this by showing how friendship is a lived, complex, and subjective experience, shaped by how individuals dwell with others in moments of vulnerability, presence, and withdrawal. In this tension between the social and the existential, a new space opens for a reenvisioning recovery as a genuine relational practice in mental health care.

### Methodological considerations

This study follows a hermeneutic phenomenological approach (van Manen, 2023). While phenomenological texts often unfold as integrative, reflective narratives that follow the inner logic of the phenomenon itself, we chose a traditional IMRAD format to communicate our insights more directly to mental health practitioners, our main audience. This format aims to maintain phenomenological depth while supporting practical use of lived-experience insights in everyday mental health care.

We included a limited number of participants, allowing for depth and closeness in analysis. However, findings may be situational and shaped by the specific context. The personal narratives offer access to meaningful, emotional, and contextual aspects of friendship, reflecting participants' understanding in their current situation. Linguistic and cultural differences between participants and the first author could have limited nuanced meanings. Participants' comfort in sharing with an unfamiliar researcher may also have affected the data's depth.

The hermeneutic interpretation assumes that the researcher’s own preunderstandings influence the analysis process (van Manen, 2023). This can provide valuable insights but also introduces subjectivity and interpretive limitations. Through repeated readings and reflective dialogue with the authors and the competence group, we have aimed to stay open to alternative interpretations and perspectives, while also recognising that certain themes or experiences have been given more emphasis than others. This is based on our understanding of which themes have the potential for phenomenological exploration.

In this study, we have developed an in-depth understanding of the lived phenomenon of friendship within the context of mental struggles and mental health work. The study’s validity depends on the ability to “touch” and gives meaning to the experience as it unfolds for those involved. van Manen (2023) emphasises that validity is demonstrated through the depth and credibility of the analysis and how the findings resonate with people’s lived experiences.

We have been concerned with research ethics in this study, with particular focus on the vulnerability factor of interviewing young people between 15 and 18 years old, who struggle with complex mental health challenges and who are receiving treatment from Youth FACT teams. According to research guidelines for recruitment of participants for the health care services, health professionals shall initiate the first contact. It was reflected upon how “gatekeepers” influence the assessment of potential participants, who, along with their vulnerability level, can consent to participate in the project. By including others in the recruitment of participants, gatekeepers’ opinions about who should participate in the project may have influenced which voices are included in the study and what knowledge we have developed.

## Concluding remarks

This study has explored lived experiences of friendship among young people and peer support workers in Youth FACT. Friendship unfolds as a complex, dynamic process without linear guarantees. Its layered meanings shifting across encounters; protective for some, burdensome for others, sometimes both within the same relationship and moment. The phenomenon under enquiry shows itself through aspects such as presence, vulnerability, movement, and freedom, offering glimpses into the genuine human experience of the phenomenon of friendship as woven into mental health challenges and recovery processes.

True to phenomenological enquiry, these insights remain partial yet invitational, calling for ongoing relational attentiveness in mental health practice. Future research should examine what relational work entails concretely and what support young people need to establish, maintain, and sustain friendships amid mental health challenges.

## Data Availability

All interviews were audio recorded and handled in accordance with national regulations (REK case no.: 934245) and institutional guidelines for research data protection (SIKT: case no.: 698643). The interview material contains rich, detailed accounts that are central to the study’s interpretations and conclusions. However, for ethical and confidentiality reasons, the full transcripts cannot be made publicly available, as participants did not consent to wider sharing of their personal narratives. Access to the data may be considered upon a reasonable request, provided robust de-identification procedures can be ensured, and any secondary use is compatible with the project's original aims and the participants’ consent.

## References

[cit0001] Act on Medical and Health Research, (LOV-2020-12-04-133). (2009). [Norwegian]. https://lovdata.no/dokument/NL/lov/2008-06-20-44

[cit0002] Åkerblom, K. B., & Mohn-Haugen, T. (2023). *Erfaringskonsulenten: funksjoner, roller og involvering [Peer support worker: functions, roles and involvement]* (1. utgave ed.). Fagbokforlaget.

[cit0003] Alsarrani, A., Hunter, R. F., Dunne, L., & Garcia, L. (2022). Association between friendship quality and subjective wellbeing among adolescents: A systematic review. *BMC Public Health*, *22*(1), 2420. 10.1186/s12889-022-14776-4 36564745 PMC9784006

[cit0004] Anthony, W. A. (1993). Recovery for mental illness: The guiding vision of mental health service system in the 1990s. *Psychosocial Rehabilitation Journal*, *16*(4), 11–34. 10.1037/h0095655

[cit0005] Beresford, P. (2019). Public participation in health and social care: Exploring the co-production of knowledge. *Frontiers in Sociology*, *3*, 41. 10.3389/fsoc.2018.00041 PMC8022568 33869395

[cit0006] Bjornestad, J., Hegelstad, WtV, Joa, I., Davidson, L., Ketil Larsen, T., Melle, I., Veseth, M., Johannessen, J. O., & Bronnick, K. (2017). With a little help from my friends” social predictors of clinical recovery in first-episode psychosis. *Psychiatry Research (Limerick)*, *255*, 209–214. 10.1016/j.psychres.2017.05.041 28578180

[cit0007] Borg, M., Karlsson, B., Kim, H. S., & McCormack, B. (2012). Opening up for many voices in knowledge construction. *Forum, Qualitative Social Research*, *13*(1). 10.17169/fqs-13.1.1793

[cit0008] Boydell, K. M., Gladstone, B. M., & Crawford, E. S. (2002). The dialectic of friendship for people with psychiatric disabilities. *Psychiatric Rehabilitation Journal*, *26*(2), 123–131. 10.2975/26.2002.123.131 12433215

[cit0009] Broersen, M., Creemers, D. H. M., Frieswijk, N., Vermulst, A. A., & Kroon, H. (2024). Effects of youth flexible assertive community treatment: Outcomes of an 18-month observational study. *Social Psychiatry and Psychiatric Epidemiology*, *59*(5), 745–758. 10.1007/s00127-023-02508-x 37280465 PMC11087363

[cit0010] Bukowski, W. M., Bagwell, C., Castellanos, M., & Persram, R. J. (2020). Friendships and Adolescence- social development in adolescence peer relationships. *The Encyclopedia of Child and Adolescent Development*. 1–11. 10.1002/9781119171492.wecad403

[cit0011] Cleary, M., Lees, D., & Sayers, J. (2018). Friendship and mental health. *Issues in Mental Health Nursing*, *39*(3), 279–281. 10.1080/01612840.2018.1431444 29465280

[cit0012] Declaration of Helsinki. (2024). [Norwegian]. https://www.forskningsetikk.no/retningslinjer/med-helse/helsinkideklarasjonen/

[cit0013] Dunbar, R. I. M. (2025). Why friendship and loneliness affect our health. *Annals of the New York Academy of Sciences*, *1545*(1), 52–65. 10.1111/nyas.15309 40047377 PMC11918532

[cit0014] FHI. (2025). Temautgave av Folkehelserapporten 2025 Barn og unges psykiske helse. [Thematic Edition of the Public Health Report 2025: Children's and Adolescents' Mental Health]. [Norwegian]. https://www.fhi.no/contentassets/b5b3603ec4794c5cb0c8651589b359f8/temautgave-barn-og-unges-psykiske-helse_2025.pdf

[cit0015] Gariépy, G., Honkaniemi, H., & Quesnel-Vallée, A. (2016). Social support and protection from depression: Systematic review of current findings in Western countries. *British Journal of Psychiatry*, *209*(4), 284–293. 10.1192/bjp.bp.115.169094 27445355

[cit0016] Gillard, S., Foster, R., White, S., Bhattacharya, R., Binfield, P., Eborall, R., Gibson, S. L., Harnett, D., Simpson, A., Lucock, M., Marks, J., Repper, J., Rinaldi, M., Salla, A., & Worner, J. (2024). Implementing peer support into practice in mental health services: A qualitative comparative case study. *BMC Health Services Research*, *24*(1), 1050. 10.1186/s12913-024-11447-5 39261915 PMC11391751

[cit0017] Gillard, S., Foster, R., White, S., Barlow, S., Bhattacharya, R., Binfield, P., Eborall, R., Faulkner, A., Gibson, S., Goldsmith, L. P., Simpson, A., Lucock, M., Marks, J., Morshead, R., Patel, S., Priebe, S., Repper, J., Rinaldi, M., Ussher, M., & Worner, J. (2022). The impact of working as a peer worker in mental health services: A longitudinal mixed methods study. *BMC Psychiatry*, *22*(1), 373. 10.1186/s12888-022-03999-9 35650562 PMC9158348

[cit0018] Gotting, E.-K., Darcy, L., Israelsson-Skogsberg, Å., Sundler, A. J., & Lalloo, E. C. (2025). Children’s experiences of living with their mental ill-health - a scoping review. *International Journal of Qualitative Studies on Health and Well-being*, *20*(1), 2501682. 10.1080/17482631.2025.2501682 40334017 PMC12064100

[cit0019] Høiseth, J. R., Hatling, T., Trane, K., Brennesvik, C., Børretzen, S., & Renskaug, I. T. (2024). FACT ung, brukermedvirkning på både system-, tjeneste-, og individnivå. [Youth FACT, User Involvement at System, Service, and Individual Levels]. *Tidsskrift for Psykisk Helsearbeid*, *21*(4), 346–357. 10.18261/tph.21.4.7

[cit0020] Johansen, M., Stuen, H. K., Brekke, E., Jensen, C. B., & Landheim, A. (2024). A qualitative study of the experiences of young people with severe mental health problems and complex needs regarding youth flexible assertive community treatment. *Frontiers in Psychiatry*, *15*, 1478345. 10.3389/fpsyt.2024.1478345 39713766 PMC11662976

[cit0021] King, A. R., Russel, T., & Veith, A. C. (2016). *Friendship and Mental Health Functioning In*. Psychology Faculty Publications.

[cit0022] Krane, V., Zubala, E., Thorning, T. H., Quintana Murci, E., & Macedo, E. (2025). Running collaborative competence groups - exploring experiences from five countries. *International Journal of Qualitative Methods*, *24*. 10.1177/16094069241306082

[cit0023] Lahad, K., & van Hooff, J. (2023). Is my best friend toxic? A textual analysis of online advice on difficult relationships. *Families, relationships and societies*, *12*(4), 572–587. 10.1332/204674321X16613283926068

[cit0024] Landheim, A., & Odden, S. r. (2020). Evaluering av FACT-team i Norge- Sluttrapport. S. I. F. Nasjonal kompetansetjeneste for samtidig rusmisbruk og psykisk lidelse (NKROP). [Evaluation of FACT Teams in Norway - Final Report. Norwegian National Competence Service for Co-occurring Substance Use and Mental Disorders]. [Norwegian]. https://rop.no/contentassets/d4f50ab571e84e24a9bd879ae14a9690/evaluering-av-fact-team-i-norge_sluttrapport_nasjonal-kompetansetjeneste-for-samtidig-rusmisbruk-og-psykisk-lidelse-nkrop.pdf

[cit0025] Leamy, M., Bird, V., Boutillier, C. L., Williams, J., & Slade, M. (2011). Conceptual framework for personal recovery in mental health: Systematic review and narrative synthesis. *British Journal of Psychiatry*, *199*(6), 445–452. 10.1192/bjp.bp.110.083733 22130746

[cit0026] Manchanda, T., Stein, A., & Fazel, M. (2023). Investigating the role of friendship interventions on the mental health outcomes of adolescents: A scoping review of range and a systematic review of effectiveness. *International journal of environmental research and public health*, *20*(3), 2160. 10.3390/ijerph20032160 36767526 PMC9915149

[cit0027] Merleau-Ponty, M. (2013). *Phenomenology of Perception*. T aylor and Francis. (Original Work Published 1945). 10.4324/9780203720714

[cit0028] Ministry of Health and Care Services. (2023). Nybrottsarbeid for unge med sammensatte hjelpebehov.[Pioneering Work for Young People with Complex Support Needs] regjeringen.no Retrieved from https://www.regjeringen.no/no/aktuelt/nybrottsarbeid-for-unge-med-sammensatte-hjelpebehov/id2962109/

[cit0029] Nord-Baade, S. (2022). FACT ung. [Youth FACT] ROP Nasjonal kompetansetjeneste for samtidig rusmisbruk og psykisk lidelse. [Norwegian].

[cit0030] Norwegian Directorate of Health. (2022). ACT-, FACT- og FACT ung-team. [ACT-, FACT- and Youth FACT] Retrieved from https://www.helsedirektoratet.no/tema/lokalt-psykisk-helse-og-rusarbeid/act-og-fact-team

[cit0031] Norwegian Directorate of Health. (2024). Vennskap og fellesskap med jevnaldrende er viktig for barn og unge. [Friendship and sense of community with peers are important for children and youth]. [Norwegian]. https://www.helsedirektoratet.no/forebygging-diagnose-og-behandling/forebygging-og-levevaner/folkehelsestatistikk-og-profiler/vennskap-og-fellesskap-med-jevnaldrende-er-viktig-for-barn-og-unge

[cit0032] Parker, J. G., & Asher, S. R. (1993). *Friendship and friendship quality in middle childhood: Links with peer group acceptance and feelings of loneliness and social dissatisfaction*. American Psychological Association.

[cit0033] Salvas, M.-C., Guimond, F.-A., Archambault, I., Vitaro, F., MacGregor, P., Cantin, S., & Robert-Mazaye, C. (2025). The role of friends in the development of children’s classroom behavioral engagement and peer acceptance in kindergarten: A dyadic approach. *Early Childhood Research Quarterly*, *73*, 27–38. 10.1016/j.ecresq.2025.06.001

[cit0034] Shah, E. N., Szwedo, D. E., & Allen, J. P. (2024). Adolescent close friendships, self-perceived social acceptance, and peer-rated likeability as predictors of wellbeing in young adulthood. *Frontiers in developmental psychology*, *2*. 10.3389/fdpys.2024.1435727

[cit0035] Skou Storm, I. M., Ulstrup Smedemark, R. A. M., Holen, M., Hybholt, L., Austin, S. F., Simonsen, E., Leamy, M., & Berring, L. L. (2025). The meaning of friendships in the mental health recovery of young Adults—A thematic analysis. *Issues in Mental Health Nursing*, *46*(1), 47–57. 10.1080/01612840.2024.2424767 39773262

[cit0036] Sommer, M. E. (2019). Support as possibility, *Lived experiences of support in the lives of young persons with mental health problems: a hermeneutic phenomenological study.* [Faculty of Health and Social science. Doctoral dissertation no. 43]. Drammen: University of South-Eastern Norway.

[cit0037] Spithoven, A. W. M., Lodder, G. M. A., Goossens, L., Bijttebier, P., Bastin, M., Verhagen, M., & Scholte, R. H. J. (2017). Adolescents' loneliness and depression associated with friendship experiences and well-being: A person-centered approach. *Journal of Youth and Adolescence*, *46*(2), 429–441. 10.1007/s10964-016-0478-2 27055683

[cit0038] Tew, J. (2004). Social perspectives in mental health: Developing social models to understand and work with mental distress, United Kingdom: Jessica Kingsley Publishers.

[cit0039] Tew, J., Ramon, S., Slade, M., Bird, V., Melton, J., & Le Boutillier, C. (2012). Social factors and recovery from mental health difficulties: A review of the evidence. *The British journal of social work*, *42*(3), 443–460. 10.1093/bjsw/bcr076

[cit0040] Topor, A., Borg, M., Di Girolamo, S., & Davidson, L. (2011). Not just an individual journey: Social aspects of recovery. *International journal of social psychiatry*, *57*(1), 90–99. 10.1177/0020764010345062 21252359

[cit0041] van Manen, M. (2016). *Researching Lived Experience: Human Science for an Action Sensitive Pedagogy (Second edition* (Second edition ed.). United Kingdom: Routledge. 10.4324/9781315421056

[cit0042] van Manen, M. (2017). Phenomenology in its original sense. *Qualitative Health Research*, *27*(6), 810–825. 10.1177/1049732317699381 28682720

[cit0043] van Manen, M. (2023). Phenomenology of practice: Meaning-giving methods in phenomenological research and writing (Second edition ed.). Routledge.

[cit0044] Viking, T., Wenzer, J., Hylin, U., & Nilsson, L. (2022). Peer support workers' role and expertise and interprofessional learning in mental health care: A scoping review. *Journal of Interprofessional Care*, *36*(6), 828–838. 10.1080/13561820.2021.2014796 35129027

